# Challenges of managing hypertension in Pakistan - a review

**DOI:** 10.1186/s40885-023-00245-6

**Published:** 2023-06-15

**Authors:** Adil Elahi, Arzina Aziz Ali, Aamir Hameed Khan, Zainab Samad, Hunaina Shahab, Namra Aziz, Aysha Almas

**Affiliations:** 1 Aga Khan University, Karachi, Pakistan; 2 Section of Cardiology, Department of Medicine, Ziauddin University, Karachi, Pakistan; 3 Section of Cardiology, Department of Medicine, Aga Khan University, Karachi, Pakistan; 4 Icahn School of Medicine, Mount Sinai, New York, NY USA; 5 Department of Medicine, Aga Khan University, Karachi, Pakistan; 6 Section of Internal Medicine, Department of Medicine, Aga Khan University, Karachi, Pakistan

**Keywords:** Hypertension, Pakistan, Review, Prevalence, Risk factors, Blood pressure

## Abstract

**Background:**

This review aims to describe existing evidence on the state of hypertension in Pakistan, including the prevalence, associated risk factors, preventive strategies, and challenges in the management of hypertension.

**Methods:**

A comprehensive literature search was conducted electronically using PubMed and Google Scholar. Using specific screening methodology, 55 articles were selected to be included.

**Results:**

We found from this extensive review that several small studies report high prevalence of hypertension but there is a lack of population based prevalence of hypertension in Pakistan. Lifestyle risk factors such as obesity, unhealthy diet, decreased physical activity, low socioeconomic status, and lack of access to care were the main associated factors with hypertension. Lack of blood pressure monitoring practices and medication non-adherence were also linked to uncontrolled hypertension in Pakistan and were more evident in primary care setups. The evidence presented is essential for delineating the burden of the disease, hence allowing for better management of this underserved population.

**Conclusion:**

There is a need for updated surveys to depict the true prevalence and management of hypertension in Pakistan. Cost-effective implementation strategies and policies at the national level are needed for both prevention and control of hypertension.

## Background

Hypertension is a leading cause of the global burden of non-communicable diseases and a significant contributor to cardiovascular disease, chronic kidney disease, and stroke [1]. Currently 25% of the world’s adult population is hypertensive, which is predicted to rise to 29% by 2025 [2]. Systematic analysis using population-based studies from 90 countries reported the prevalence of hypertension as 31.5% in low-and-middle-income countries (LMIC), compared to 28.5% in high-income countries [3]. Not only is the prevalence of hypertension growing in developing countries, but the age of onset of cardiovascular disease (including hypertension) is decreasing [2], and thus contributes to about two-thirds of the mortality attributed to hypertension [4]. A study from the second National Diabetes Survey of Pakistan (NDSP) 2016, concluded an alarming prevalence of hypertension in Pakistan that urges immediate attention [5].

Hypertension exists alongside cardiovascular disease, and several behavioral and socio-demographic characteristics are seen to be linked with both [6]. Cardiovascular disease (CVD) is consistently evolving and will be the foremost cause of mortality in 2030 [7]. Despite the seriousness of cardiovascular diseases, minimal attention is directed towards the prevention of its risk factors, including hypertension. Pakistan is a country in South Asia and is the fifth most populous country in the world [8]. The healthcare system of Pakistan consists of a private sector (70%) and a public sector (30%). Only 27% of the population benefits from full healthcare coverage, whereas 73% depend on out-of-pocket payments [9, 10]. Economic and political instability is a leading cause for inadequate preventive and control strategies, thus accelerating rates of hypertension and cardiovascular diseases in this country [11]. This review aims to describe the existing state of hypertension in Pakistan, including its prevalence, associated factors for control of hypertension, and evidence on management of hypertension.

## Main text

**Fig. 1:**
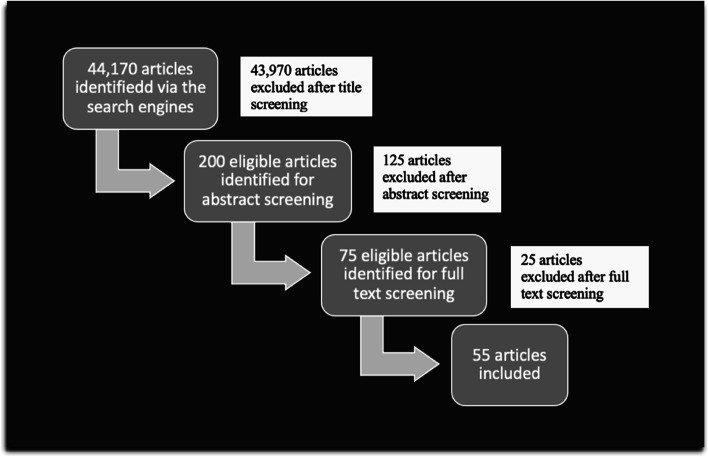
Study flow chart—articles and bibliography

This review includes articles from PubMed and google scholar search engines and bibliography yielded 44,170 articles out of which 55 articles were selected using the screening methods shown in Fig. 1. Table 1 lists some of the main studies included in this review.

**Table 1:** Summary of studies on hypertension in Pakistan

Project Title	Authors	Study Design	Sample Size	Objectives	Results
Perceptions and Practices towards Medication Non-Adherence among Hypertensive Patients: An Observational Study	Khan M N, Soomro N, Ashraf T, et al.	Cross-sectional, single-center study	200 patients	To evaluate medication adherence and the contributing factors of HTN along with the assessment of common perceptions and practices related to its management.	• No association between medication adherence and educational level • Perceptions like ‘BP is mostly controlled’ and ‘BP medication necessity’ was also positively associated with adherence
Prevalence and contributing risk factors for hypertension in urban and rural areas of Pakistan; a study from second National Diabetes Survey of Pakistan (NDSP) 2016–2017	Basit et al.	Community-based epidemiological survey	10,800 adults	To assess the prevalence and its associated risk factors for hypertension in urban and rural areas of Pakistan	• Prevalence of hypertension in urban and rural areas was 44.3% and 46.8%, respectively • Age, female gender, married individuals, positive family history of hypertension, diabetes, and obesity were highly significantly associated with hypertension
Risk factors assessment for hypertension in a squatter settlement of Karachi	Siddiqui et al.	Cross-sectional survey	327 adults	To assess the risk factors for hypertension in adults (age more than 15 years) in a squatter settlement of Karachi	• Hypertensives were 9.7 times more likely to be diabetic • no significant difference was noticed for education, smoking status, family history and occupation
Prevalence and Risk Factors of Hypertension Among Children Attending Outpatient Department of a Tertiary Care Hospital in Karachi	Bilal et al.	Cross-sectional study	1000 children	To identify the risk factors and the prevalence of HTN among the pediatric age group attending the OPD (out-patient department) of a tertiary care hospital in Karachi	• Higher prevalence of HTN and pre-HTN among children of 4–7 years of age • Significant association of BMI, family history of HTN, and high-fat diet intake with the prevalence of HTN
Children in South Asia have higher body mass-adjusted blood pressure levels than white children in the United States: a comparative study	TH Jafar et al.	Cross-sectional study	18,135 children	To determine the prevalence of high blood pressure in children in Pakistan and study the correlates of systolic and diastolic blood pressure in South Asian children	• The mean BMI (SD) was significantly lower in children in Pakistan • High blood pressure in Pakistani children 5 to 14 years old was 12.2% compared to 5% in US
Factors associated with hypertension in Pakistan: A systematic review and meta-analysis	Riaz et al.	Meta-analysis	30 cross-sectional and 7 case-control studies	To conduct a thorough systematic review and meta-analysis of the literature to make valid inferences about the risk factors of hypertension in Pakistan and provide greater consistency and validity to the previous results	• Adults aged 30–60 years who were married, in urban areas, high incomes, used tobacco, positive family history, and positive comorbidities were positively associated with hypertension
Good knowledge about hypertension is linked to better control of hypertension; a multicenter cross-sectional study in Karachi, Pakistan	Almas et al.	Cross-sectional study across 3 centers	447 adults	To elucidate the knowledge about hypertension and compare the knowledge of those with uncontrolled hypertension and controlled hypertension	• Total mean (SD) Knowledge score was 20.97(4.93) out of a maximum score of 38 • Sufficient knowledge about hypertension in patients has been associated with greater medication adherence
Hypertension-related Knowledge and Its Relationship with Blood Pressure Control in Hypertensive Patients Visiting a Semi-private Tertiary-care Charity Hospital in Karachi, Pakistan	Nadeem, Mohammad Khurram et al.	cross-sectional study	355 patients	To ascertain the knowledge of hypertension and other sociodemographic variables and their impact on controlling blood pressures in the hypertensive population belonging to the low socioeconomic strata	• No significant association was observed between the levels of knowledge of hypertension and gender, blood pressure (BP) status, professional activity, and age groups
Determinants of Uncontrolled Hypertension in Rural Communities in South Asia-Bangladesh, Pakistan, and Sri Lanka	Tazeen H Jafar et al.	Cross-sectional study	1,718 individuals aged ≥ 40 years	To determine the cross-country variation, and the factors associated with uncontrolled BP among adults treated for hypertension in rural South Asia	• 58.0% had uncontrolled BP: 52.8% in Bangladesh, 70.6% in Pakistan, and 56.5% in Sri Lanka • The odds of uncontrolled BP were significantly higher in individuals with lower wealth index, not married, higher urine albumin-to-creatinine ratio, lower estimated glomerular filtration rate, lower adherence to antihypertensive, and in Pakistan vs. Sri Lanka
Better physician-patient communication; an important milestone in control of hypertension, a multicenter study from Karachi, Pakistan	Almas A, Bhamani F, Khan AH.	Control trial study	3 tertiary care hospitals	To compare physician encounter score in patients with controlled and uncontrolled hypertension.	• Mean physician encounter score in uncontrolled hypertensive was 7.25 ± 2.64 versus 7.83 ± 2.22 in controlled hypertensive. • Patient-physician encounter is an important milestone in control of hypertension in hypertensive patients and directly translates into better adherence to antihypertensives in these patients.
Non-Adherence to Prescribed Antihypertensives in Primary, Secondary and Tertiary Healthcare Settings in Islamabad, Pakistan: A Cross-Sectional Study	Mahmood S, Jalal Z, Hadi MA, Orooj H, Shah KU	Cross-sectional study	741 patients	To assess the prevalence and predictors of non-adherence to antihypertensive medication among patients with hypertension attending various healthcare settings in Islamabad, Pakistan	• Binary regression analysis revealed that old age, being educated, free medical care, treatment duration, number of medications, presence of any comorbidity and blood pressure control were significantly associated with good adherence • Main reasons for non-adherence to prescribed antihypertensive medication: ‘don’t feel need for regular use’ 24.7%), ‘Carelessness’ (13.4%) and ‘adverse effects’ (11.2%)
Medication Adherence and Its Association with Health Literacy and Performance in Activities of Daily Livings among Elderly Hypertensive Patients in Islamabad, Pakistan	Saqlain M, Riaz A, Malik MN, et al.	Cross-sectional survey-based study	262 adults	To investigate medication adherence and its associated factors among Pakistani geriatric hypertensive patients	• Self-reported moderate and good subjective health, adequate health literacy, and independence in performing activities of daily living were found to be independent predictors of medication adherence among older hypertensive patients
Out of pocket (OOP) cost of treating hypertension in Karachi, Pakistan	Aslam N, Shoaib MH, Bushra R, et al.	Cross-sectional prevalence-based study	350 adults	To determine the cost of treating hypertension form the patient’s perspective (out of pocket cost) in Karachi, Pakistan	• The average treatment cost of stage 1 was observed to be significantly lower (*p* = 0.006) than the cost of stage 2 HTN • Significant difference was observed in the average cost of drugs, consultancy fees and laboratory tests, but no variation was seen in cost of transport and loss of productivity through absenteeism from work
Awareness of hypertension among patients attending primary health care centre and outpatient department of tertiary care hospital of Karachi	Ashfaq T, Anjum Q, et al.	Cross sectional survey	202 patients	To compare awareness of hypertension among patients attending Primary Health Care Centre (PHC) and outpatient department (OPD) of a tertiary care hospital of Karachi.	• Majority of the patients attending tertiary care OPD (80%) and 56% from PHC group believed that hypertension could lead to cardiovascular disease (CVD) • 61% from tertiary care OPD group and 31% of PHC group said antihypertensives were taken only for few months • 77% of patient of tertiary OPD were not doing exercise and not avoiding oily and heavy food
Current trends in treatment of hypertension in Karachi and cost minimization possibilities	Hussain IM, Naqvi BS, Qasim RM, Ali N.	Three-pronged approach was used. Two randomized stratified surveys.	100 doctors 400 patients	To find drug usage trends in Stage I Hypertensive Patients without any compelling indications, deviations of current practices from evidence based antihypertensive therapeutic guidelines, and cost minimization opportunities.	• Majority of patients were prescribed Beta Blockers (33%), followed by Angiotensin Converting Enzyme (18%), Calcium Channel Blockers (13%) and Diuretics (8%) • For most cost-effective drug, 29% of the doctors opined Beta Blockers, followed by Angiotensin Converting Enzymes inhibitors (18%) and diuretics (12%)
Can community-based interventions control hypertension in developing countries? What is the evidence from Pakistan?	Majeed F, Kamal AK.	Cluster randomized controlled trial, in 12 randomly selected communities in Karachi	4023 between 5–39 years 1341 patients 40 years or older	To determine the impact of family-based home health education (HHE) on blood pressure in children, and adults at a community level over a 2 year follow up period	• In participants, aged 5–39 years analyses showed significant blood pressure reductions in the intervention arm which received home health education • In hypertensive patients over 40 years of age, there was a significant 10 mmHg improvement in systolic blood pressure in patients who were assigned to both home education and General Practitioner education
Enhanced hypertension care through private clinics in Pakistan: a cluster randomised trial	Khan MA, Khan N, Walley JD, et al.	A two-arm cluster randomised controlled trial	1138 patients in 13 clusters	To assess whether enhanced care at urban private clinics resulted in better control of hypertension, cardiovascular disease (CVD) risk factors, and treatment adherence	• mean intervention outcome was -25.2 mmHg; mean control outcome was -9.4 mmHg; and mean control–intervention difference was 15.8 • The findings support the scaling of an integrated CVD–hypertension care intervention in urban private clinics in areas lacking public primary care in Pakistan

### Prevalence of hypertension in Pakistan

#### Hypertension in adults

We still await a large population-based survey to record the prevalence of hypertension in the existing 216 million population in Pakistan. According to the last population-based National Health Survey of Pakistan (NHSP), nearly 18.9% of people in Pakistan above 15 years were hypertensive, with a higher prevalence in urban populations than rural population, and in men more than women [12]. Despite the lack of population-based data on hypertension prevalence, multiple studies have been conducted to determine the prevalence of hypertension sporadically in multiple provinces and cities of Pakistan. The WHO Stepwise approach to surveillance (STEPS) was conducted in the two large provinces, Sindh and Punjab, and reported a prevalence of stage 1 hypertension (SBP > 140 mmHg and DBP > 90 mmHg) to be 37% and stage II hypertension (SBP > 160 mmHg and DBP > 100 mmHg) to be 15.9% [12]. More recently in the National Diabetes Survey of Pakistan (NDSP) 2016–2017, a large community-based epidemiological survey in all four provinces (*n* = 10,834), the prevalence of hypertension was 46.2% [5]. Other small-scale urban studies reported prevalence ranging from 15 to 29% [13, 14]. Furthermore, the high rates of uncontrolled hypertension in Pakistan, which represented more than one-fourth of all patients in the medical emergency department, inevitably lead to hypertensive crises in 56.3% of these patients [15].

#### Hypertension in children and adolescents

Hypertension is not just limited to adults. There is a disproportionately higher blood pressure (BP) burden in South Asian children with relatively unclear reasons [16]. Jafer et al. reported that increased obesity in children was associated with elevated levels of BP in South Asia [17, 18]. These differences persisted even with lower Body Mass Index (BMI) thresholds, and children at or above these thresholds were twice as likely to have an increase in BP than those not centrally obese or overweight [18]. Furthermore, elevated blood pressure during childhood is considered a predictor of hypertension during adulthood [19]. A study comparing blood pressure in South Asian children versus American children, found that 12.2% of Pakistani children between ages 5 and 14 years have high blood pressure; significantly higher than children in the United States (*p* < 0.001) [20]. This finding was observed despite Pakistani children having lower BMIs. Bilal M et al. conducted a study on one thousand children who visited an outpatient clinic in 2019 in urban Karachi and reported a 25% prevalence of Hypertension (HTN) in children aged between 4 and 7 years, and pre-HTN in 10% [21]. It also showed a strong association between HTN in children and weight, family history, and high-fat diet, concluding that large-scale screening of young children is required to report the prevalence of elevated blood pressures.

In summary, the prevalence of hypertension is high in Pakistan, with almost 2/3rds of presenting patients suffering from it. From 2000 to 2010, the prevalence of hypertension decreased by 2.6% in high-income countries but increased by 7.7% in low/middle–income countries [22]. A study reported an increasing prevalence of 46.2% in Pakistan in 2016–2017. Reviews done in other low-middle income countries in 2017 showed a similar increase with time, such as 40.6% in India [23], 40.7% in Bangladesh [24], and 34.0% in Zambia [25]. The main reasons for this rise are disparities in awareness, treatment, and control rates, due to the lack of resources, education, and funds in low-middle income countries [26]. Our study reflects this data and Pakistan is shown to be one of the low-middle income countries continuously struggling with these health disparities.

### Risk factors for hypertension in Pakistan

A meta-analysis of 37 studies extracted 46 factors that were significantly associated with hypertension in the Pakistani population [27]. The three most frequently reported factors were old age (15), gender (14), and BMI (9) [27]. A national Non-Communicable Disease (NCD) survey for risk factors in two major provinces of Pakistan found a high prevalence of HTN, with the respondents having either low fruit and vegetable consumption (96.5%), low physical activity (46%), being overweight (26.3%), or obesity (14.9%) [13].

#### Obesity

The association of BMI with hypertension has been reported in multiple studies, specifically a BMI greater than 23 kg/m^2^ [28]. Akram J et al. noted obesity prevalence in Pakistan was 70% and had significant association with hypertension (HTN) (*p* = 0.0003) [29]. They also found obesity was higher among Pakistani women than men. The childhood obesity epidemic has caused the global prevalence of (HTN) in children to increase from 2 to 4% [21]. However, there is limited data on obese children and HTN in Pakistan.

#### Unhealthy diet

The Pakistan Adolescents Schools Survey 1 reported that over 80% of adolescents in Pakistan had unhealthy diets [30]. Studies in high-income populations have shown that diets high in red meat, fatty food, and sweet desserts are associated with an increase in blood pressure, whereas diets with high amounts of fruits, vegetables, whole grains, and lean meat are associated with lower blood pressure [31]. However, Asian populations have different environmental and genetic factors, and few studies have been done in this population, assessing the role of dietary patterns on hypertension. A study on the Pakistani population reported a decrease in HTN with yogurt and seafood dietary patterns, but no statistically significant effect between high-fat and sugar dietary patterns and hypertension [32]. The same study also did not find a significant effect of high fruit and vegetable diet on blood pressure reduction in the Pakistani population.

#### Physical inactivity

A WHO study showed that 82.8% of boys and 87.3% of girls aged 13‐15 are not sufficiently active [33]. 54.3% of Pakistani adolescents are physically inactive according to another study [30]. Schools without playgrounds, female gender, and lack of parental support for sports correlated with physical inactivity among the students [34]. In a cross-sectional survey in Karachi, physical activity was significantly associated with a lower risk of hypertension [35]. Men were more likely to be more active, as culturally they have greater access to the outdoor facilities compared to the women. These findings highlight the need for increased physical activity and its relevance and contributions to hypertension.

An assessment of 21 countries reports that the main reason contributing to these risk factors could be the reinforcement of sedentary lifestyles through new technology, increased office jobs, conversion of agricultural lands to factories, and transportation facilities that make physical activity more difficult [36].

Literature reports established risk factors for hypertension, including lifestyle behaviors, physical inactivity, unhealthy diet, obesity, low socioeconomic level, and smoking [36]. Results from this review are consistent with literature, with a particular emphasis on obesity, unhealthy diet, and decreased physical activity, all of which in turn can also be attributed to low socioeconomic level. Global reviews have displayed that in high-income countries, the decline in blood pressure has occurred despite rising BMI, whereas both BMI and blood pressure are rising in most LMIC [26, 37, 38]. This can be explained by enhanced social and economic development, including the availability of nutritious food options, better housing infrastructure, and importance given to early childhood and adolescent nutrition in high income countries [39–42]. LMIC must look to higher income countries to adopt strategies used to reduce risk factors. An Example of addressing specific risk factors is the reduction of salt intake in the Republic of South Africa, where legislation was passed on the limitation of the upper limit of sodium content in processed foods [43]. Lifestyle changes are a crucial and actionable risk factor and must be emphasized by both the medical professionals and government agencies of Pakistan, where counseling and education can improve the compliance and sustainability of healthy habits.

### Blood pressure monitoring practice in Pakistan

According to the European Society of Cardiology (ESC) 2018 guidelines, auscultatory or oscillometric semiautomatic or automatic sphygmomanometers are the preferred method for measuring BP [44]. Oscillometric devices are recommended for office BP as they give the best results. However, due to the enhanced cost requirements, it cannot be recommended as the primary method in resource-poor countries [45]. Low-income countries such as Pakistan continue to use the mercury sphygmomanometer despite its multiple problems. Studies in this setting report wide variation in blood pressure monitoring in clinic settings with white coat hypertension reported in 16% of patients [46]. Moreover Shahab et al. introduced the concept of Post-clinic BP, as it had a good correlation with ambulatory BP and may be considered a more reliable method of monitoring BP [47].

### Control rate of hypertension in Pakistan

Hypertension control is poor in most Asian countries [48]. In primary care settings, developed countries like Canada have a higher percentage of patients with controlled hypertension (65%) compared to developing nations like Pakistan, where the control rate is 6% [49]. According to the National Health Survey of Pakistan, 17.9% of adults over 15 years had hypertension, and from those only 3% had hypertension under control [50]. The proportion of patients with controlled hypertension differs among developed and developing countries, specifically between primary and tertiary settings. A cross-sectional study from rural areas of Pakistan, Bangladesh, and Sri Lanka showed that low adherence to antihypertensive therapy was one of the critical factors independently associated with high odds of uncontrolled BP [51]. Moreover, most individuals in each country were taking only one antihypertensive medication, due to affordability and availability. Rates of control are better in tertiary care settings. A study coordinated in three different tertiary care centers in Karachi reported that 72.3% of non-diabetic hypertensive patients had controlled BP, defined as an average BP of less than 140/90 mmHg [50]. Higher age, male gender, and greater than 10 years of formal education were significant factors leading to more controlled hypertension. A study from 2019 at a single-center tertiary center in Karachi reported that 62.5% of patients took antihypertensive medication daily, and more than half of that studied population had their BP controlled [52]. Like other studies, this study discovered that gender, smoking, additional comorbidities, and medication compliance were significant factors associated with uncontrolled hypertension.

### Challenges in controlling hypertension

The nonexistence of screening programs, problems in accessing health care facilities, lack of communication between provider and patient, and high treatment costs are substantial barriers to the hypertension control in Pakistan [53]. A qualitative study conducted at Aga Khan Hospital, Karachi, found that lack of insight about disease seriousness, deficiency in knowledge, health care system limitations, inadequate health education, financial constraints, religious belief, all acted as barriers to proper control of NCDs including hypertension [54]. In contrast, patients with higher education levels, family history of HTN, fear of premature death, and previous comorbidities were more likely to perform self-care behavior than newly diagnosed patients [54].

#### Medication nonadherence

Adherence is defined as the extent to which patients can follow the recommendations for prescribed treatments. Patients may become non-adherent when they decide not to fill their prescriptions, take less than the prescribed treatment, use their medication at the wrong time, or discontinue treatment prematurely [55]. Level of education, number of medications, and presence of comorbidities were significantly associated with medication adherence in Pakistan [56]. A cross-sectional study among individuals treated for hypertension in rural South Asia, and urban Pakistan, reported an independent relationship between low wealth index and poor adherence to medications with a higher likelihood of uncontrolled blood pressure [57, 58]. Another cross-sectional study evaluating drug compliance and associated factors in patients from Karachi found that patients stopped taking medication when they traveled away from home and when symptoms were controlled [59]. The study also found that male gender, duration of hypertension of more than 5 years, and regular BP monitoring were associated with higher rate of compliance.

#### Lack of universal health care (UHC)

UHC means that all individuals and communities receive the health services they are entitled to without suffering financial hardship. It includes the full spectrum of essential, quality health services, from health promotion to prevention, treatment, rehabilitation, and palliative care across the life course [60]. Unfortunately, in Pakistan, the majority of the healthcare cost burden is incurred by the household, as 60% of the total health expenditures come from out-of-pocket (OOP) expenditures, with the largest (> 52%) share of OOP expenditure being medication [61]. A study in Pakistan, showed that blood pressure and diabetes medication were a substantial portion of household expenditures, competing with other necessities such as food and education [61]. The percentage of uncontrolled hypertension was higher in rural Pakistan compared to Bangladesh and Sri Lanka, due to scarce use of antihypertensive medications in Pakistan with a lack of universal health coverage, and where more than 80% of spending on medications is out-of-pocket (OOP) [51]. In Karachi, a study conducted to assess the OOP cost of treatment of hypertension found that the total cost for stage I and II hypertension was 217,869.7PKR and 17,545,457.6 PKR, respectively, demonstrating that cost alone causes an increased burden on the average-earning man, ultimately resulting in medication nonadherence [62].

#### Lack of knowledge about hypertension

A study conducted at three tertiary centers in Karachi, Pakistan, found that the knowledge score in hypertensive patients was poor and even lower in those with uncontrolled hypertension [63]. The mean physician encounter score was seen to be higher in controlled hypertensive patients compared to those with uncontrolled hypertension (*p* < 0.05), depicting better physician-patient communication results in sufficient medication adherence and ultimately improved control of hypertension [63]. Statistically significant differences in knowledge between patients with controlled and uncontrolled hypertension was observed, with regards to the meaning of hypertension, target systolic and diastolic blood pressures, hypertension being a lifelong disease with lifelong treatment, asymptomatic hypertension, lifestyle changes, and high blood pressure associated with aging [50]. Another study comparing knowledge and attitude toward hypertension between patients in primary care settings and patients in tertiary care hospitals found marked differences between the two groups, in importance of medication adherence and the role of diet, exercise, and weight reduction on BP management [64]. The difference between the patients in these two settings was explained by their education level, as a significant number of patients visiting Primary Healthcare Center (PHC) had no, or minimal education [64]. There can be multiple reasons for the lack of knowledge about hypertension in our population. Lack of patient education by the physician, lack of health education through media, and lack of proper infrastructure and resources are a few [50]. A study conducted in Lahore, Pakistan, to evaluate the effect of educational intervention on the control of BP found that after the intervention there was an increase in medication adherence, and lower systolic and diastolic blood pressure [65]. Hence, it is necessary to increase patient knowledge, and the physician plays a critical role in this.

### Evidence on management of hypertension in Pakistan

The widely recommended treatment of initial hypertension involves the use of Thiazide Diuretics, Angiotensin-Converting Enzyme Inhibitors (ACEIs), or Calcium Channel Blockers (CCBs) [66]. The Joint National Committee (JNC) guidelines recommend the use of thiazide diuretics for most patients [67], whereas Pakistan Hypertension League’s guidelines, which resemble the National Institute for Health and Care Excellence (NICE) guidelines, recommend that patients younger than 55 years of age should be treated with ACEIs and those older than 55 years or the black population, should be treated with CCB and diuretics [68]. Diuretics are recommended due to their safety profile, cost-effectiveness, and cardiovascular protection [68]. Beta-blockers are now not recommended for the initial treatment of hypertension due to their diabetogenic potential [68]. But Pakistani doctors did not adhere to pharmacologic treatment guidelines as beta-blockers were prescribed most extensively (33%), followed by ACEI (18%) and CCBs (13%), whereas thiazide diuretics were hardly used [68]. This is despite hydrochlorothiazide from a national company being the most cost-effective drug for a patient [68]. Furthermore, up to four times more expensive branded generics were commonly prescribed and would translate into increased cost of treatment [68]. In another study, only 3.7% of doctors talked about the Dietary Approaches to Stop Hypertension (DASH) diet with their patients or were advising salt reduction to hypertensive patients. Furthermore, smoking cessation counseling to hypertensive patients was mentioned by only 4.3% of doctors [69]. A recent systematic review on prescribing antihypertensive patterns in Pakistan, from a total of 26 studies from 2000 to 2018, concluded that only ten studies utilized national or international guidelines a better understanding of a clinician’s perception and practice of hypertension management guidelines in LMICs is needed [70].

### Intervention trials on hypertension from Pakistan

Limited intervention trials on hypertension management have been done in Pakistan so far. A cluster randomized control trial conducted in 12 communities of Karachi showed that among the population between the ages of 5 and 39 years, significant systolic and diastolic blood pressure reduction occurred in the intervention group which received home health education (HHE) in a public health setting [71]. The trial also demonstrated that among adult hypertensive patients, there was a greater proportion of patients achieving controlled blood pressure and a greater drop witnessed in systolic BP in patients who received both HHE and general practitioner education combined than those who received either HHE or general practitioner education or none [71]. Thus, this trial highlighted the importance of combined community education and caretaker (GP) education in controlling hypertension in Pakistan. A two-arm cluster randomized control trial in a private health sector in Punjab exhibited enhanced management of hypertension in the intervention group that received a clinical guide with standardized prescription, adherence support, and lifestyle modifications [72]. The trial also found a significant increase in the proportion of patients with controlled hypertension and higher treatment adherence in the intervention arm than in the control arm. Both these studies highlight the importance of public-private partnership to implement hypertension control at a mass level. Table 1 summarizes the existing studies on hypertension conducted in Pakistan.

In summary, there are many barriers to the successful management of hypertension in resource-limited settings and a lack of trials on how to overcome them. There is a need for changes in policymaking to allow for the management of hypertension through cost-effective strategies, preventative health promotions, providing affordable treatment, and updating therapeutic guidelines [19, 61, 62]. The Community-Based Intervention for Managing Hypertension in Rural South Asia study by Jafer et al. has shown success of a multicomponent intervention based on home visits by trained government community health workers and led to a greater reduction in blood pressure than usual care among adults with hypertension [17]. This model has also been tested in other South Asian countries including Sri Lanka and Bangladesh.

In other studies from Pakistan, the need for medical advancements and research and improvement in healthcare delivery is shown to be of extreme importance to achieve control of hypertension. This would involve focusing on preventative care with regular BP screening, effective communication by the providers, standardized treatment approaches, providing cost-effective treatment, and ensuring accountability [73]. Pakistan’s neighboring country, India, has reported that awareness, treatment, and control are lower in populations with primary or no education, reflecting a combination of low socioeconomic status, which may influence access to care, lack of knowledge of the sequelae of uncontrolled hypertension, and differing values concerning the importance of blood pressure control [74]. Ultimately treatment coverage and effectiveness remain low in low-income settings [75]. Socioeconomic inequality in LMIC affects the use of drugs and secondary prevention medicines [76].

Better developed health systems, such as in the United States of America where there is more socioeconomic stability, are more effective in managing HTN [77, 78]. There is an increased need for government and policymakers to make available the resources necessary to build on the existing healthcare infrastructure. This means building both physical and human resource capacity, as well as the use of several non-traditional hypertension screening methods. An example of such a model has been successful in several LMICs through programs spearheaded by the Global Alliance for Chronic Disease [79]. Further trials on hypertension management are necessary for establishing guidelines and recommendations catered to low- and middle-income countries.

The strength of this review is that it is a comprehensive search of databases to ensure all relevant articles were identified. Potential bias was reduced by having the authors independently scan through the search output and extract relevant data. Some limitations are that the probability that health outcomes such as hypertension may influence reports of smoking, drinking habits and other lifestyle factors, and not vice versa, cannot be disregarded. Most studies included in this review are cross sectional, and their design does not affirm causality. Studies included in this review varied in design, population, assessment of risk factors, and timeframe of the sample collected, hindering the ability to compare results. Moreover, the different methods of identification of hypertensive patients could misjudge the true prevalence, especially when hypertension was self-reported. There is a large amount of heterogeneity in data, due to which a meta-analysis could not be done.

## Conclusion

Pakistan is shown to be one of the low-middle income countries continuously struggling with health disparities including hypertension control. Although the prevalence of hypertension is proven to be high in Pakistan, more robust population-based survey prevalence figures are lacking. There is a need for updated surveys to depict the true prevalence and control rate of hypertension in Pakistan and implementation strategies for hypertension control across the country.

Implementation of studies like the Community-Based Intervention for Managing Hypertension in Rural South Asia across the country are study needed for prevention and management of hypertension across Pakistan. Digital health technology must be tested and implemented in blood pressure control models in future, as these actions have shown proven benefits in other countries.

Lifestyle risk factors and low socioeconomic level are the leading barriers to HTN management in Pakistan. LMIC must look to higher income countries to adopt strategies used to reduce risk factors. Lifestyle changes are a crucial and actionable risk factor and must be emphasized by both medical professionals and government agencies of Pakistan, where counseling and education can improve compliance and sustainability of healthy habits.

There is a need for changes in policymaking to allow for the management of hypertension through cost-effective strategies, preventative health promotions, providing affordable treatment, updating therapeutic guidelines and ensuring the implementation of guidelines by healthcare professionals. The categorization of Pakistan as a LMIC, and thus having weak policymaking and implementation, is the root of most health disparities in the country.

## Data Availability

Data sharing is not applicable to this article as no datasets were generated or analyzed during the study.

## Abbreviations

HTN: Hypertension
ACEIs: Angiotensin-Converting Enzyme Inhibitors
CCBs: Calcium Channel Blockers
WHO STEPS: World Health Organization Stepwise Approach to surveillance
NDSP: National Diabetes Survey of Pakistan
CVD: Cardiovascular disease
LMIC: Low-and-middle-income countries
BP: Blood Pressure
BMI: Body Mass Index
ESC: European Society of Cardiology
NCD: Non-Communicable Disease
UHC: Universal Health Care
OOP: Out-of-pocket
JNC: Joint National Committee
NICE: National Institute for Health and Care Excellence
DASH: Dietary Approaches to Stop Hypertension
PHC: Primary Healthcare Center
HHE: Home Health Education

## References

[1] Yao Q, Liu C, Zhang Y, Xu L (2019). Health-related quality of life of people with self-reported hypertension: a national cross-sectional survey in China. Int J Environ Res Public Health.

[2] Kearney PM, Whelton M, Reynolds K, Muntner P, Whelton PK, He J (2005). Global burden of hypertension: analysis of worldwide data. Lancet (London, England).

[3] Mohsen IM (2018). Hypertension in developing countries: a major challenge for the future. Curr Hypertens Rep.

[4] Prabhakaran D, Jeemon P, Ghosh S, Shivashankar R, Ajay VS, Kondal D (2017). Prevalence and incidence of hypertension: results from a representative cohort of over 16,000 adults in three cities of South Asia. Indian Heart J.

[5] Basit A, Tanveer S, Fawwad A, Naeem N, NDSP Members (2020). Prevalence and contributing risk factors for hypertension in urban and rural areas of Pakistan; a study from second National Diabetes Survey of Pakistan (NDSP) 2016–2017. Clin Exp Hypertens.

[6] Shafi ST, Shafi T (2017). A survey of hypertension prevalence, awareness, treatment, and control in health screening camps of rural central Punjab, Pakistan. J Epidemiol Glob Health.

[7] Cardiovascular diseases. Available from: https://www.who.int/health-topics/cardiovascular-diseases#tab=tab_1. Cited 2022 Dec 26.

[8] Pakistan - Wikipedia. Available from: https://en.wikipedia.org/wiki/Pakistan. Cited 2022 Dec 25.

[9] Hassan A, Mahmood K, Allah Buksh H. Healthcare system of Pakistan. Int J Adv Res Publ. 2017;1(4):170-3.

[10] WHO EMRO | Health service delivery | Programmes | Pakistan. Available from: https://www.emro.who.int/pak/programmes/service-delivery.html. Cited 2022 Dec 26.

[11] Barolia R, Sayani AH (2017). Risk factors of cardiovascular disease and its recommendations in Pakistani context. J Pak Med Assoc.

[12] Rafique I, Saqib MAN, Munir MA, Qureshi H, Rizwanullah KS, Khan SA (2018). Prevalence of risk factors for noncommunicable diseases in adults: key findings from the Pakistan STEPS survey. East Mediterr Health J.

[13] Siddiqui H, Anjum Q, Omair A, Usman J, Rizvi R, Ashfaq T (2005). Risk factors assessment for hypertnesion in a squatter settlement of Karachi. J Pak Med Assoc.

[14] Ishtiaq S, Ilyas U, Naz S, Altaf R, Afzaal H, Muhammad AS (2017). Assessment of the risk factors of hypertension among adult & elderly group in twin cities of Pakistan. J Pak Med Assoc.

[15] Almas A, Ghouse A, Iftikhar AR, Khursheed M (2014). Hypertensive crisis, burden, management, and outcome at a tertiary care center in Karachi. Int J Chronic Dis.

[16] Fowokan A, Punthakee Z, Waddell C, Rosin M, Morrison KM, Gupta M (2019). Multifactorial correlates of blood pressure in South Asian children in Canada: a cross-sectional study. BMJ Open.

[17] Jafar TH, Gandhi M, de Silva HA, Jehan I, Naheed A, Finkelstein EA (2020). A community-based intervention for managing hypertension in rural South Asia. N Engl J Med.

[18] Jafar TH (2009). Children, obesity, and high blood pressure: Asian populations at high risk. Am J Hypertens.

[19] Jafar TH, Islam M, Hatcher J, Hashmi S, Bux R, Khan A (2010). Community based lifestyle intervention for blood pressure reduction in children and young adults in developing country: cluster randomised controlled trial. BMJ.

[20] Jafar TH, Islam M, Poulter N, Hatcher J, Schmid CH, Levey AS (2005). Children in south Asia have higher body mass-adjusted blood pressure levels than white children in the United States. Circulation.

[21] Bilal M, Haseeb A, Saeed A, Saeed A, Ghaffar P (2020). Prevalence and risk factors of hypertension among children attending out patient department of a tertiary care hospital in Karachi. Cureus.

[22] Bloch MJ (2016). Worldwide prevalence of hypertension exceeds 13 billion. J Am Soc Hypertens.

[23] Abariga SA, Khachan H, Al Kibria GM (2020). Prevalence and determinants of hypertension in India based on the 2017 ACC/AHA guideline: evidence from the India National Family Health Survey. Am J Hypertens.

[24] Islam JY, Zaman MM, Haq SA, Ahmed S, Quadir ZA (2018). Epidemiology of hypertension among Bangladeshi adults using the 2017 ACC/AHA hypertension clinical practice guidelines and joint National Committee 7 guidelines. J Hum Hypertens.

[25] Goma FM, Mwewa B, Tembo GK, Kachamba M, Syatalimi C, Simweemba C (2019). May measurement month 2017: blood pressure screening results from Zambia-Sub-Saharan Africa. Eur Heart J Suppl.

[26] Zhou B, Perel P, Mensah GA, Ezzati M (2021). Global epidemiology, health burden and effective interventions for elevated blood pressure and hypertension. Nat Rev Cardiol.

[27] Riaz M, Shah G, Asif M, Shah A, Adhikari K, Abu-Shaheen A (2021). Factors associated with hypertension in Pakistan: a systematic review and meta-analysis. PLoS One.

[28] Jafar TH, Chaturvedi N, Pappas G (2006). Prevalence of overweight and obesity and their association with hypertension and diabetes mellitus in an Indo-Asian population. Can Med Assoc J.

[29] Akram J, Rehman HR, Muneer F, Hassan S, Fatima R, Khan TM (2021). Hypertension and obesity: a cross-sectional study. Eur J Med Heal Sci.

[30] Khuwaja AK, Khawaja S, Motwani K, Khoja AA, Azam IS, Fatmi Z (2011). Preventable lifestyle risk factors for non-communicable diseases in the Pakistan Adolescents Schools Study 1 (PASS-1). J Prev Med Public Health.

[31] Mansoori S, Kushner N, Suminski RR, Farquhar WB, Chai SC (2019). Added sugar intake is associated with blood pressure in older females. Nutrients.

[32] Safdar N, Bertone-Johnson E, Cordeiro L, Jafar T, Cohen N (2015). Dietary patterns and their association with hypertnesion among Pakistani urban adults. Asia Pac J Clin Nutr.

[33] World Health Organization. Regional Office for the Eastern Mediterranean. Country factsheet insufficient physical activity. Pakistan; 2015. https://apps.who.int/iris/handle/10665/204252.

[34] Jabeen I, Zuberi R, Nanji K (2018). Physical activity levels and their correlates among secondary school adolescents in a township of Karachi, Pakistan. J Pak Med Assoc.

[35] Ajani K, Gowani A, Gul R, Petrucka P (2021). Levels and predictors of self-care among patients with hypertension in Pakistan. Int J Gen Med.

[36] Lim SS, Vos T, Flaxman AD, Danaei G, Shibuya K, Adair-Rohani H (2012). A comparative risk assessment of burden of disease and injury attributable to 67 risk factors and risk factor clusters in 21 regions, 1990–2010: a systematic analysis for the Global Burden of Disease Study 2010. Lancet.

[37] Zibara V, Costanian C, Al Haddad N, Kilani H, Tohme F, Bahous SA (2021). Epidemiology and management of hypertension among refugees in the Middle East: a review of the literature. J Hum Hypertens.

[38] Bentham J, Di CM, Bilano V, Bixby H, Zhou B, Stevens GA (2017). Worldwide trends in body-mass index, underweight, overweight, and obesity from 1975 to 2016: a pooled analysis of 2416 population-based measurement studies in 128·9 million children, adolescents, and adults. Lancet (London, England).

[39] Ezzati M, Zhou B, Bentham J, Di Cesare M, Bixby H, Danaei G (2018). Contributions of mean and shape of blood pressure distribution to worldwide trends and variations in raised blood pressure: a pooled analysis of 1018 population-based measurement studies with 88.6 million participants. Int J Epidemiol.

[40] Micha R, Khatibzadeh S, Shi P, Andrews KG, Engell RE, Mozaffarian D. Global, regional and national consumption of major food groups in 1990 and 2010: a systematic analysis including 266 country-specific nutrition surveys worldwide on behalf of the Global Burden of Diseases Nutrition and Chronic Diseases Expert Group. Nutri Open. 2015;5:1-23. 10.1136/bmjopen-2015-008705 PMC4593162 26408285

[41] Lewington S, Li L, Sherliker P, Guo Y, Millwood I, Bian Z (2012). Seasonal variation in blood pressure and its relationship with outdoor temperature in 10 diverse regions of China: the China Kadoorie Biobank. J Hypertens.

[42] Bentham J, Di Cesare M, Stevens GA, Zhou B, Bixby H, Cowan M, et al. A century of trends in adult human height. Elife. 2016;5. Available from: https://pubmed.ncbi.nlm.nih.gov/27458798/. Cited 2022 Dec 26. 10.7554/eLife.13410 PMC4961475 27458798

[43] Peters SAE, Dunford E, Ware LJ, Harris T, Walker A, Wicks M (2017). The sodium content of processed foods in South Africa during the introduction of mandatory sodium limits. Nutrients.

[44] Williams B, Mancia G, Spiering W, Agabiti Rosei E, Azizi M, Burnier M (2018). 2018 ESC/ESH guidelines for the management of arterial hypertension. Eur Heart J.

[45] Siddique S, Hameed Khan A, Shahab H, Zhang Y, Chin Tay J, Buranakitjaroen P (2021). Office blood pressure measurement: a comprehensive review. J Clin Hypertens.

[46] Godil SS, Tabani H, Khan AH, Almas A (2011). White coat hypertension is not a benign entity: a cross-sectional study at a tertiary care hospital in Pakistan. J Pak Med Assoc.

[47] Shahab H, Khan HS, Almas A, Tufail M, Kazmi KA, Khan AH (2018). The Post Clinic Ambulatory Blood Pressure (PC-ABP) study correlates Post Clinic Blood Pressure (PCBP) with the gold standard Ambulatory Blood Pressure. BMC Res Notes.

[48] Chia Y, Kario K, Turana Y, Nailes J, Tay JC, Siddique S (2020). Target blood pressure and control status in Asia. J Clin Hypertens.

[49] Nadeem MK, Mari A, Iftikhar S, Khatri A, Sarwar T, Patel MJ (2019). Hypertension-related knowledge and its relationship with blood pressure control in hypertensive patients visiting a semi-private tertiary-care charity hospital in Karachi, Pakistan. Cureus.

[50] Almas A, Godil SS, Lalani S, Samani ZA, Khan AH (2012). Good knowledge about hypertension is linked to better control of hypertension; a multicentre cross sectional study in Karachi, Pakistan. BMC Res Notes.

[51] Jafar TH, Gandhi M, Jehan I, Naheed A, de Silva HA, Shahab H (2018). Determinants of uncontrolled hypertension in rural communities in South Asia—Bangladesh, Pakistan, and Sri Lanka. Am J Hypertens.

[52] Khan MN, Soomro N, Ashraf T, Naseeb K, Kumar R, Bhatti U (2019). Perceptions and practices towards medication non-adherence among hypertensive patients: an observational study. Cureus.

[53] Legido-Quigley H, Naheed A, de Silva HA, Jehan I, Haldane V, Cobb B (2019). Patients’ experiences on accessing health care services for management of hypertension in rural Bangladesh, Pakistan and Sri Lanka: a qualitative study. PLoS One.

[54] Gowani A, Ahmed HI, Khalid W, Muqeet A, Abdullah S, Khoja S (2016). Facilitators and barriers to NCD prevention in Pakistanis-invincibility or inevitability: a qualitative research study. BMC Res Notes.

[55] Hugtenburg J, Vervloet M, van Dijk L, Timmers L, Elders. Definitions, variants, and causes of nonadherence with medication: a challenge for tailored interventions. Patient Prefer Adherence. 2013;675-82. 10.2147/PPA.S29549 PMC3711878 23874088

[56] Almas A, Hameed A, Ahmed B, Islam M (2006). Compliance to antihypertensive therapy. J Coll Physicians Surg Pak.

[57] Almas A, Patel J, Ghori U, Ali A, Edhi AI, Khan MA (2014). Depression is linked to uncontrolled hypertension: a case–control study from Karachi, Pakistan. J Ment Health.

[58] Saqlain M, Riaz A, Malik MN, Khan S, Ahmed A, Kamran S (2019). Medication adherence and its association with health literacy and performance in activities of daily livings among elderly hypertensive patients in Islamabad, Pakistan. Medicina (B Aires).

[59] Ali K, Adil SO, Soomro N, Bibi A, Kalam S (2018). Drug compliance and its associated factors among hypertensive patients in Pakistan: a cross-sectional study. Hosp Pharm.

[60] Universal Health Coverage. Available from: https://www.who.int/health-topics/universal-health-coverage#tab=tab_1. Cited 2022 Dec 26.

[61] Datta BK, Husain MJ, Fatehin S (2019). The crowding out effect of out-of-pocket medication expenses of two major non-communicable diseases in Pakistan. Int Health.

[62] Aslam N, Shoaib MH, Bushra R, Farooqi FA, Zafar F, Ali H (2018). Out of pocket (OOP) cost of treating hypertension in Karachi, Pakistan. Pak J Pharm Sci.

[63] Almas A, Bhamani F, Khan AH (2014). Better physician-patient communication; an important milestone in control of hypertension, a multicenter study from Karachi, Pakistan. J Coll Physicians Surg Pak.

[64] Ashfaq T, Anjum Q, Siddiqui H, Shaikh S, Vohra EA (2007). Awareness of hypertension among patients attending primary health care centre and outpatient department of tertiary care hospital of Karachi. J Pak Med Assoc.

[65] Amer M, Rahman N, Nazir SR, Raza A, Riaz H, Sultana M (2018). Impact of pharmacist’s intervention on disease related knowledge, medication adherence, HRQoL and control of blood pressure among hypertensive patients. Pak J Pharm Sci.

[66] Al-Makki A, DiPette D, Whelton PK, Murad MH, Mustafa RA, Acharya S (2022). Hypertension pharmacological treatment in adults: a world health organization guideline executive summary. Hypertension.

[67] Schwartz GL, Sheps SG (1999). A review of the sixth report of the Joint National Committee on Prevention, Detection, Evaluation, and Treatment of High Blood Pressure. Curr Opin Cardiol.

[68] Hussain IM, Naqvi BS, Qasim RM, Ali N (2015). Current trends in treatment of hypertension in Karachi and cost minimization possibilities. Pak J Med Sci.

[69] Mohydin B, Siddique S, Mumtaz K (2021). Arterial hypertension management trends in Pakistan; cross-sectional observational study of a sample of Pakistani doctors. J Hypertens.

[70] Arshad V, Samad Z, Das J, Almas A, Rashid N, Virani SS (2021). Prescribing patterns of antihypertensive medications in low- and middle-income countries: a systematic review. Asia Pac J Public Health.

[71] Majeed F, Kamal AK (2012). Can community based interventions control hypertension in developing countries? What is the evidence from Pakistan?. J Pak Med Assoc.

[72] Khan MA, Khan N, Walley JD, Khan SE, Hicks J, Sheikh FI (2019). Enhanced hypertension care through private clinics in Pakistan: a cluster randomised trial. BJGP Open.

[73] Dzau VJ, Balatbat CA (2019). Future of hypertension. Hypertension.

[74] Zaman MJ, Patel A, Jan S, Hillis GS, Raju PK, Neal B (2012). Socio-economic distribution of cardiovascular risk factors and knowledge in rural India. Int J Epidemiol.

[75] Mills KT, Bundy JD, Kelly TN, Reed JE, Kearney PM, Reynolds K (2016). Global disparities of hypertension prevalence and control: a systematic analysis of population-based studies from 90 countries. Circulation.

[76] Murphy A, Palafox B, O’Donnell O, Stuckler D, Perel P, AlHabib KF (2018). Inequalities in the use of secondary prevention of cardiovascular disease by socioeconomic status: evidence from the PURE observational study. Lancet Glob Health.

[77] Moran AE, Odden MC, Thanataveerat A, Tzong KY, Rasmussen PW, Guzman D (2015). Cost-effectiveness of hypertension therapy according to 2014 guidelines. N Engl J Med.

[78] Axon RN, Bradford WD, Egan BM (2009). The role of individual time preferences in health behaviors among hypertensive adults: a pilot study. J Am Soc Hypertens.

[79] Vedanthan R, Bernabe-Ortiz A, Herasme OI, Joshi R, Lopez-Jaramillo P, Thrift AG (2017). Innovative approaches to hypertension control in low- and middle-income countries. Cardiol Clin.

